# Tunable electronic and photoelectric properties of Janus group-III chalcogenide monolayers and based heterostructures

**DOI:** 10.1038/s41598-024-61373-z

**Published:** 2024-05-10

**Authors:** Yipeng Zhao, Qiaolai Tan, Honglai Li, Zhiqiang Li, Yicheng Wang, Liang Ma

**Affiliations:** 1 College of Physics and Electronic Engineering, Hengyang Normal University, Hengyang, 421008 China; 2 School of Physics and Electronic Electrical Engineering, Xiangnan University, Chenzhou, 423000 China; 3 College of Physics Science and Technology, Hebei University, Baoding, 071002 People’s Republic of China

**Keywords:** Condensed-matter physics, Structural properties, Two-dimensional materials

## Abstract

Janus group-III chalcogenide monolayers and based heterostructures with breaking vertical structural symmetry offer additional prospects in the upcoming high-performance photoelectric devices. We studied the geometrical, electronic, and photoelectric properties of Janus group-III chalcogenide monolayers and heterostructures. The most energy favorable stacking design of ten vertical heterostructures are considered. The results showed that the Janus Se-In-Ga-S and S-In-Ga-Se monolayers exhibit semiconducting characteristics with the band gaps of 1.295 eV and 1.752 eV, respectively. Furthermore, the different stacking configurations and surface termination at interface can realize the transition of band alignment between type I and type II due to the interlayer coupling. Moreover, we systematically investigated the photoelectric properties of Janus group-III chalcogenide heterostructures and predicated an optimized power conversion efficiency of 16.2%. These findings can aid in comprehending the customized characteristics of Janus group-III chalcogenide heterostructures, offering theoretical guidance for creating innovative photoelectric devices.

## Introduction

Since the successful exfoliation of graphene, two-dimensional (2D) materials have received worldwide interest due to their astonishingly physical properties and potential implementation in next-generation electronic and photoelectric devices^1–5^. Among various 2D materials, group-III monochalcogenides (MX, M=Ga, In; X=S, Se) have received more attention in recent years due to their remarkable mechanical, electronic, and optical properties^6–8^. The MX monolayers has a honeycomb lattice structure and stacking in the order of X-M-M-X. To date, various MX monolayers had been successfully synthesized, and it exhibited high carrier mobility, good metal contacts, high thermal stability, and the absence of dangling bond^9–11^. All these merits make MX monolayers promising for photoelectric devices and improve the motivation to design heterostructures based on these materials^12–14^. However, MX monolayers display relatively large indirect band gaps with 2.0–4.0 eV, resulting a poor absorption in the visible light spectrum^15,16^. Thus, it is very important to find suitable means to realize effective tunable of electronic structure and optical properties in MX monolayer for its practical application^17–21^.

Generally, the atom structure symmetry plays a crucial role in the determination of electronic properties for ultrathin materials^22–25^. Due to its lattice asymmetry, the Janus monolayers has an intrinsic built-in electric field in the vertical direction compare with traditional MXs, which can separate the charge carriers and enhance the electron-phonon interaction^26–28^. In addition, Janus MX monolayers showed distinct physical properties such as excellent absorption coefficient, high charge carrier mobility, and rapid separation of photogenerated carriers, which gives them potential for photovoltaic and photoelectric applications^29,30^. For instance, the Janus In_2_SSe monolayer possesses an indirect-direct bandgap transition due to the broken vertical symmetry^31^. Bui et al. systematically studied the structural, electronic, and optical properties of Janus Ga_2_XY and In_2_XY (Y = S, Se, Te) monolayers, and compared the acquired electronic band gaps with their binary analogs^32^. Zhong et al. have been predicted the dynamic stability of Janus Ga_2_XY monolayer, and the phonon dispersions confirmed that the monolayers can exist as a freestanding structure^33^. Furthermore, Ahmad et al. found that the band gaps of XGaInY monolayer extend from 0.74 to 2.66 eV, and the light absorption coefficients were greater than 10^4^ cm^−1^ in the visible and ultraviolet region^34^.

In addition, van der Waals (vdW) heterojunctions opens the way for new promising applications as they maintain the advantages of each monolayer and introduce new exciting properties due to the interlayer coupling^35–37^. Inspired by the traditional 2D vdW heterojunctions, several attempts have been proposed to various heterostructures based on Janus monolayers to explore their novel properties^38–40^. To date, numerous MX-based vdW heterostructures have been studied, such as In_2_SeTe/Ga_2_STe and SeGa_2_Te/SeIn_2_Se^41,42^. More interestingly, the MX-based vdWs heterostructures show the power conversion efficiency (PCE) up to 13.17%, while it further boosted to 21% in Janus In_2_SeTe/Ga_2_STe lateral heterostructures^42^. In addition, the Janus-In_2_STe/InSe lateral heterostructures exhibit a high optical absorption coefficient of 8×10^5^ cm^−1^ in visible light zone^43^. Particularly, the combination of Janus monolayers gives birth to versatile heterostructures with magnificent properties such as excellent optical, tunable electrical contact properties, and etc^44–46^.

Herein, we put forward first-principles simulations to examine the structure, electronic, photoelectric properties of Janus MX monolayers and MX-based heterostructures. Herein, we first evaluated the structural parameters and cohesive energy of Janus MX monolayers, and further investigated their electronic band structure and optical properties. Next, we construct ten vertical heterostructures of Janus MX with different stacking configurations, and investigated the electronic band structure and band diagrams of the most stable configurations. In addition, the enhanced light absorption of heterostructures is presented and the PCE of the heterostructures was evaluated. Our results establish that Janus MX and based heterostructures would be the probable candidates for electronic and photoelectric applications.

## Computational details

All the first-principles computations within the framework of density-functional theory (DFT) were carried out by DS-PAW software. The Device Studio program provides several functions for performing visualization and modeling. We chose the generalized gradient approximation (GGA) in the Perdew–Burke–Ernzerhof (PBE) formalism to describe the exchange–correlation potential^47–49^. A vacuum thickness of 20 Å (for single layer material) and 40Å (for heterojunction) are built to avoid the interactions between adjacent layers. The Monkhorst-Pack k-point meshes of 9×9×1 is used for geometric optimization and electronic structure calculation of Janus monolayers and heterojunction. A plane wave basis set with a cutoff energy of 500 eV was employed for plane wave expansion. In our calculations, the force and energy parameters of atomic relaxation are set as 0.01 eV/Å and 10^−8^ eV. To confirm the origin of the catalytic activity of vertical heterojunction, density of states (DOS) calculations and Bader charge analysis were performed. In the process of heterojunction optimization and computation, the DFT-D3 method with Grimme correction is adopted to describe the long-range van der Waals interactions^50^. Additionally, the more accurate Heyd–Scuseria–Ernzerhof (HSE06) hybrid functional was employed to check the reliability of the band structure^51^.

## Results and discussion

**Figure 1:**
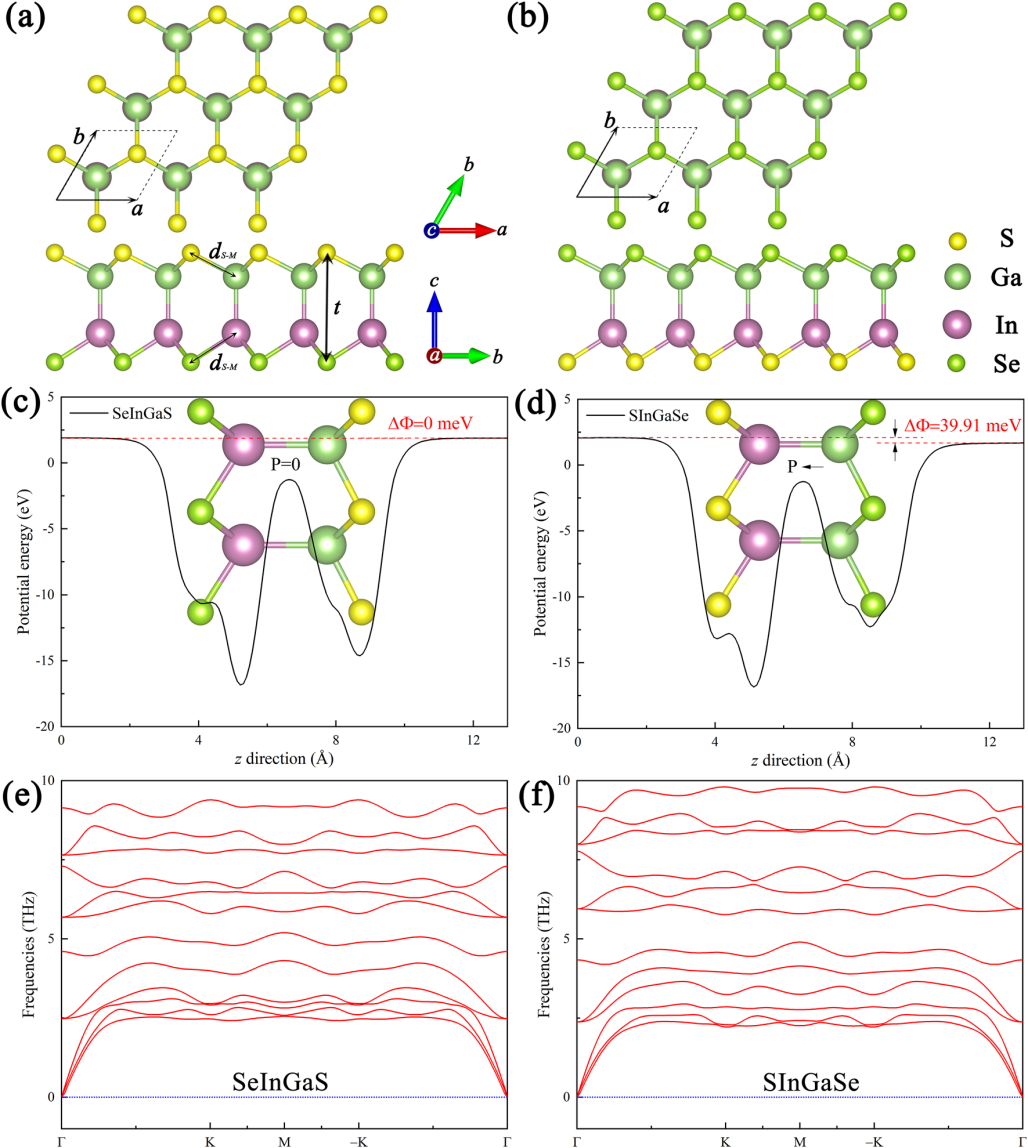
(**a**,**b**) Top view and side view of the Janus Se-In-Ga-S and S-In-Ga-Se monolayers. The average potential energy of the Janus Se-In-Ga-S (**c**) and S-In-Ga-Se (**d**) monolayers. The phonon spectrum of Janus Se-In-Ga-S (**e**) and S-In-Ga-Se (**f**) monolayers.

Figure 1a,b depicts the top and side view of atomic crystal structure in Janus Se-In-Ga-S and S-In-Ga-Se monolayers. Clearly, the Janus MX monolayer is made up of X-M-M’-X’ configuration with broken mirror symmetry in the vertical direction. The optimized lattice constant, bond lengths and thicknesses after relaxation are listed in Table 1. The Ga-In bond lengths in the different Janus monolayers remain particularly unchanged, being similar to Ga–Ga and In–In bond lengths in traditional MX monolayer. In addition, the Bader charge analysis shows that the charge is transferred from metal atoms to chalcogenide atoms, as shown in Fig. 1c,d. For instance, the Ga and In atoms lose 0.773e and 0.706e for the Janus Se-In-Ga-S monolayer, while the Se and S atoms receive 0.662e and 0.818e, respectively. Actually, the Bader charge transfer is associated with the type of element and the bond length between the metal and chalcogenide atoms^52^. The bond length of M-S (M-Se) is 2.42 Å (2.62) in Janus Se-In-Ga-S and 2.52 Å (2.50) in S-In-Ga-Se monolayers, respectively. As can be seen in Fig. 1e,f, the calculated phonon spectra of Janus Se-In-Ga-S and S-In-Ga-Se monolayers shows no imaginary frequency, indicating its dynamic stability. The chalcogenide atoms attract electrons from metal atoms since the chalcogenide atoms are more electronegative^53^. The different arrangements of metal and chalcogen atoms have noticeable effects on the charge transfer and electronic properties.

**Table 1:** Calculated lattice constant (*a* and *b*), layer spacing (*d*), S-M and Se-M chemical band length(*d*_*S-M*_, *d*_*Se-M*_), total thickness (*t*) and electronic band gap (*E*_*g*_) using different exchange correlation functionals for monolayer Janus Se-In-Ga-S and SInGaS.

Monolayer Janus	*a* (Å)	*b *(Å)	*d*_*S-M*_ (Å)	*d*_*Se-M*_ (Å)	*t* (Å)	*E*_*g*_^*PBE*^ (eV)	*E*_*g*_^*HSE06*^ (eV)
Se-In-Ga-S	3.82	3.82	2.42	2.62	5.03	1.29	2.14
S-In-Ga-Se	3.84	3.84	2.52	2.50	5.00	1.74	2.61

**Figure 2:**
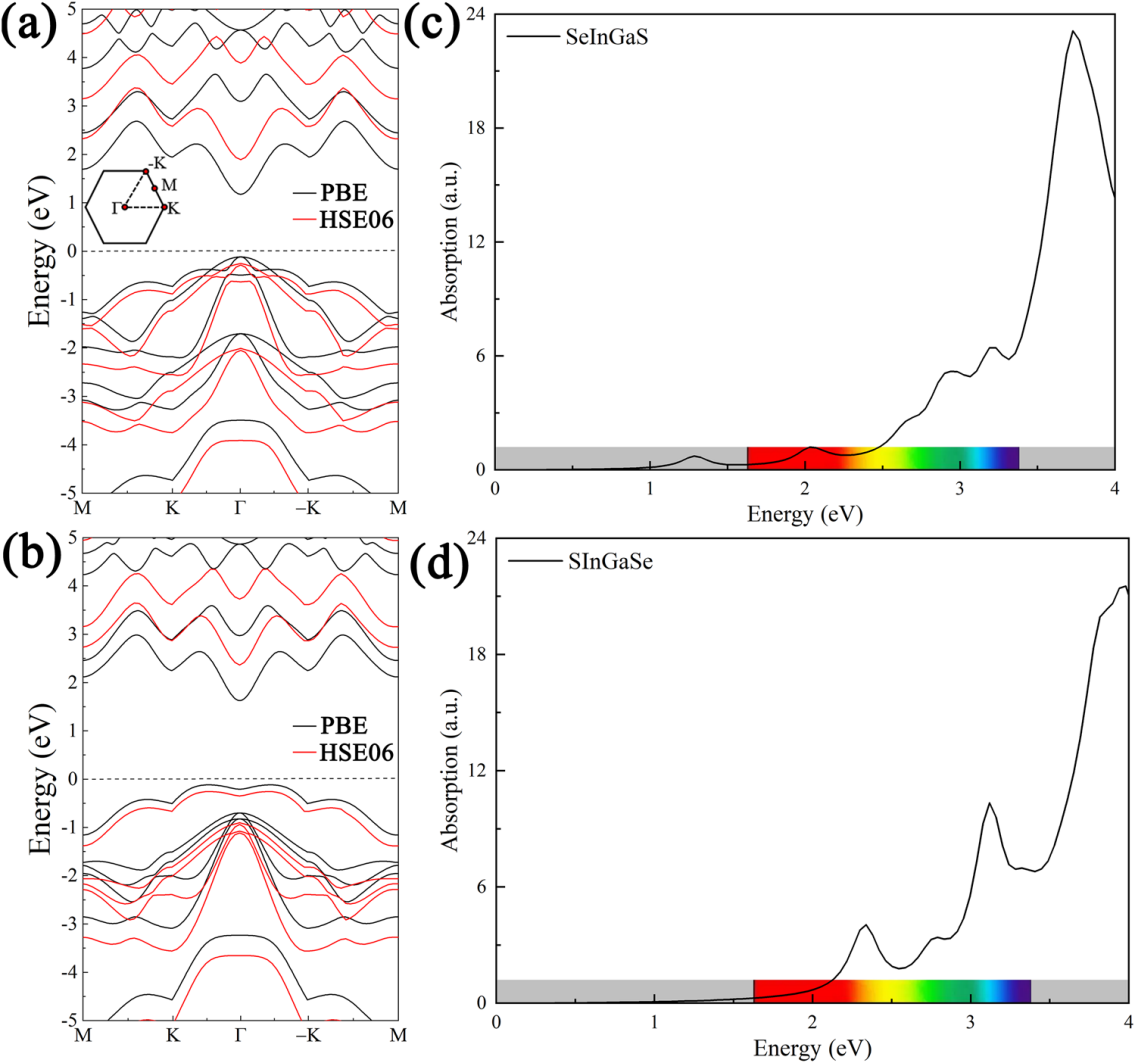
(**a**,**b**) The band structure calculated using PBE and HSE06 methods for the Janus Se-In-Ga-S and S-In-Ga-Se monolayers. The calculated optical absorption coefficients of the Janus Se-In-Ga-S (**c**) and S-In-Ga-Se (**d**) monolayers.

The electronic band structures of Janus Se-In-Ga-S and S-In-Ga-Se monolayers were calculated at the PBE and HSE06 level along the high symmetry points of M–K–Γ–K–M, as shown in Fig. 2a and b. Clearly, the Janus Se-In-Ga-S and S-In-Ga-Se monolayers preserve the semiconducting character, with the band gaps of 1.295 eV (2.14 eV) and 1.752 eV (2.61 eV) with the PBE (HSE06) functional, respectively. The PBE bandgap is always lower than the HSE06 bandgap. The band structure calculated using HSE06 hybrid functional are consistent with the results calculated using PBE functional for the type of band gap^34^. The results show that the Se-In-Ga-S monolayers exhibit direct gaps with their valence band maximum (VBM) and conduction band minimum (CBM) points located at the Γ-point, while the S-In-Ga-Se monolayers are indirect band gap. Actually, the CBM of Se-In-Ga-S monolayers is main contributed by the *s* and *p*_z_ orbitals of Ga atoms and small contribution from *s* and *p*_*z*_-orbitals of In atoms, and the VBM is mainly determined by the *p*_*x*_ and *p*_*y*_ orbitals of Se atoms. The CBM of S-In-Ga-Se monolayers is organized by the *s* orbitals of Ga atoms and small contribution from *s* orbitals of In and Se atoms, and the VBM is mainly determined by the *p*_*z*_ orbitals of S and Se atoms and small contribution from *p*_*z*_ orbitals of In and Ga atoms. The orbital-resolved band structures of Janus Se-In-Ga-S and S-In-Ga-Se monolayers show in Fig. S1. The band gap of MX can be effectively tuned by constructing the Janus structure, which provides an effective method to tailor the photoelectric properties of MX monolayers. In addition, Fig. 2c,d depicts the optical absorption coefficients of the Janus Se-In-Ga-S and S-In-Ga-Se monolayers as a function of photon energy. Obviously, the monolayers show considerable visible light and near-ultraviolet absorption, which can be ascribed to the enhanced hybridization states near the VBM and the CBM. The excellent absorption coefficients in the visible region indicates that the Janus Se-In-Ga-S and S-In-Ga-Se monolayers are suitable for further applications.

**Figure 3:**
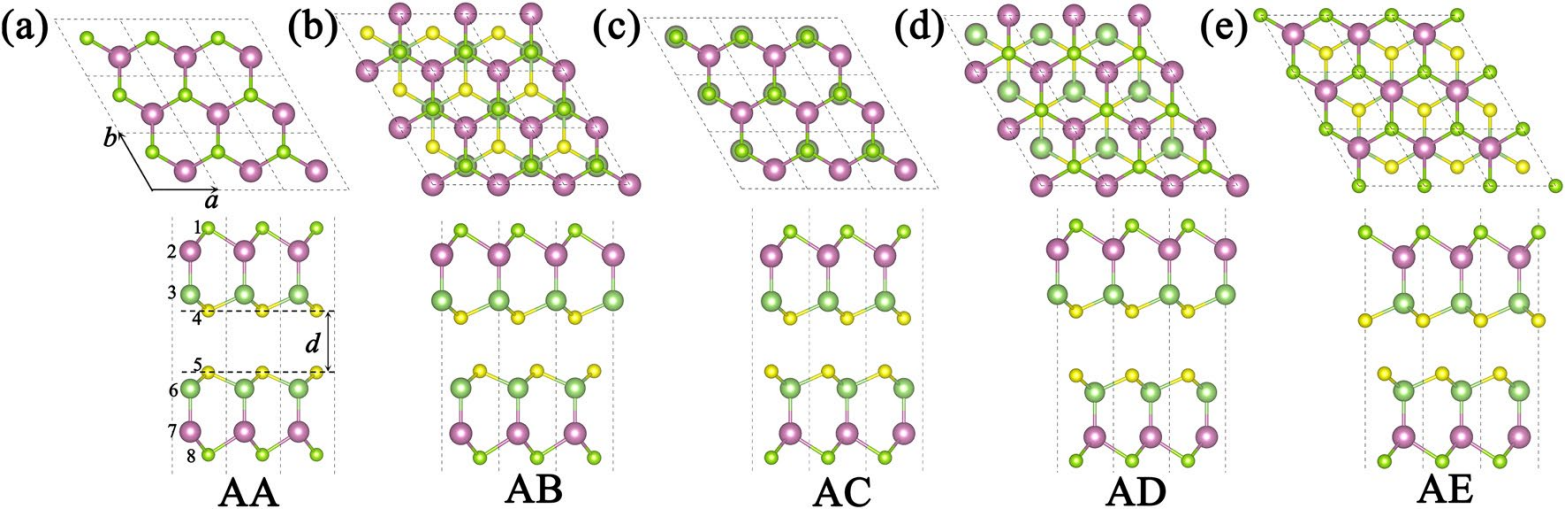
Top view and side view of the five models of Janus heterostructures.

The structure and stability properties of the heterostructures based on Se-In-Ga-S and S-In-Ga-Se were investigated. Since both the Janus Se-In-Ga-S and S-In-Ga-Se monolayers are mirror symmetry broken with two different terminated surfaces, here we construct totally 10 vertical configurations of heterostructure. Such as SSA, SSB, and SSC configurations, their commonality lies in the interlayer S element attached to each other (defined as SS), while their difference lies in the arrangement of metal element In and Ga. The atomic arrangement order and structural abbreviation of vertical heterojunctions are shown in Table 2. Furthermore, to check the energy stability of heterostructures, we calculated the binding energy: $E_{coh} = E_{{SGaInSe{/}SeGaInS}} - E_{SGaInSe} - E_{SeGaInS}$, where $E_{{SGaInSe{/}SeGaInS}}$ is the total energy of the heterostructure,$E_{SGaInSe}$ and $E_{SeGaInS}$ are the total energy of the isolated Se-In-Ga-S and S-In-Ga-Se monolayer, respectively. For SSA configurations, there have five different stacking patterns, shown in Fig 3. For all the configurations, the binding energies are all negative, and the most energy favorable stacking pattern are also listed in Table S1. The AB stacking has the smallest binding energies, which is most favorable stacking pattern. The calculated lattice parameter, equilibrium interlayer distance of favorable stacking pattern are also summarized in Table 3.

**Table 2:** SS: Interlayer S element is attached to S element; SeSe: Interlayer Se element is attached to Se element; A type: Two layers of material Ga element adhered to Ga element; B type: Two layers of material Ga element adhered to In element; B’ type: Two layers of material In element adhered to Ga element; C type: Two layers of material In element adhered to In element.

	Element order	Type									
		SSA	SSB	SSC	SeSeA	SeSeB	SeSeC	SSeB	SSeA	SSeC	SSeB’
First layer	1	Se	Se	Se	S	S	S	Se	Se	Se	Se
	2	In	In	Ga	In	In	Ga	In	In	Ga	Ga
	3	Ga	Ga	In	Ga	Ga	In	Ga	Ga	In	In
	4	S	S	S	Se	Se	Se	S	S	S	S
Second layer	5	S	S	S	Se	Se	Se	Se	Se	Se	Se
	6	Ga	In	In	Ga	In	In	In	Ga	In	Ga
	7	In	Ga	Ga	In	Ga	Ga	Ga	In	Ga	In
	8	Se	Se	Se	S	S	S	S	S	S	S

**Table 3:** Calculated lattice constant (*a* and *b*), interlayer distance (*d*), electronic band gap (*E*_*g*_^*PBE*^), conduction band offset (CBO) and power conversion efficiency (PCE) for different heterostructures.

Heterostructure	*a* (Å)	*b* (Å)	*d* (Å)	*E*_*g*_^*PBE*^ (eV)	CBO (eV)	PCE (%)
SSA	3.82	3.82	3.07	1.103	0.176	12.8
SSB	3.83	3.83	3.03	1.176	0.604	6.8
SSC	3.84	3.84	3.01	1.304	0.426	12.9
SeSeA	3.84	3.84	3.22	1.238	0.492	10.3
SeSeB	3.83	3.83	3.18	1.039	0.271	16.2
SeSeC	3.82	3.82	3.16	1.035	0.272	12.7
SSeB	3.82	3.82	3.11	1.065	0.217	14.6
SSeA	3.83	3.83	3.14	1.042	0.241	13.6
SSeC	3.83	3.83	3.08	1.164	0.613	6.4
SSeB’	3.84	3.84	3.11	1.157	0.575	6.9

**Figure 4:**
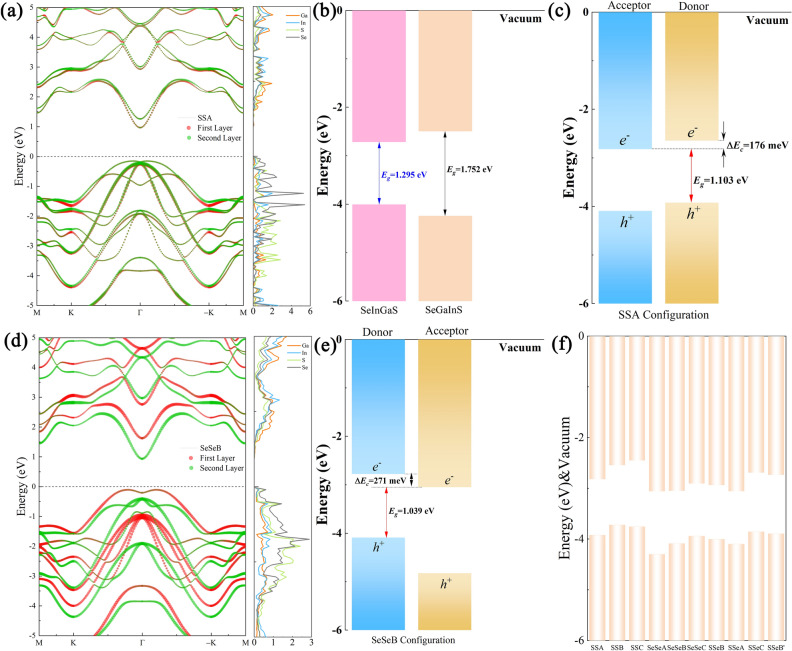
The band structure and DOS of the 2D Janus group-III chalcogenide for SSA (**a**) and SeSeB (**d**) configuration. (**b**) Band arrangement of Janus Se-In-Ga-S and S-In-Ga-Se monolayers. Band arrangement of SSA (**c**) and SeSeB (**e**) configuration. (**f**) Band arrangement of Janus heterostructures.

The electronic band structures of the vertical heterostructure with different stacking pattens are shown in Fig. 4a,d and Figs. S2, S3. The phonon spectra curves of SSA and SeSeB configuration are described in Fig. S4, and shows no imaginary frequency, which further verify the dynamical stabilities of these configuration. The heterostructures are indirect band gap semiconductors with the CBM located at Γ point and the VBM located near the Γ point of the Brillouin zone. The band gap of heterostructure with SSA configuration is 1.103 eV, which is smaller to the pristine Janus Se-In-Ga-S and S-In-Ga-Se monolayers. Clearly, the CBM of the heterostructures for SSA configuration is mainly contributed by the electronic states from the top layer, while the VBM is dominated by the bottom layer. Therefore, the type II band alignment of heterostructures can be found and the conduction band offset (CBO) between acceptor and donor is 0.176 eV, as shown in Fig. 4b,c. While for SeSeB configuration, the CBM is contributed by the top Se-In-Ga-S layer, and the VBM is dominated by the bottom S-In-Ga-Se layer, as shown in Fig. 4d,e. The band gap and CBO of SeSeB configuration are 1.039 eV and 0.271 eV, respectively. Typically, the type-II band alignment at interface allows the electrons and holes to be separated in different materials, which can effectively reduce the recombination of electron-hole. Actually, the band alignment is strongly dependent on the surface termination owing to the intrinsic internal electric field of the Janus monolayer^54^. The band gap of other different configurations is list in Fig. 4f. The tunable interface coupling and band alignment provide an ideal platform for promoting the effective separation of photogenerated carriers and facilitating the quantum efficiency.

**Figure 5:**
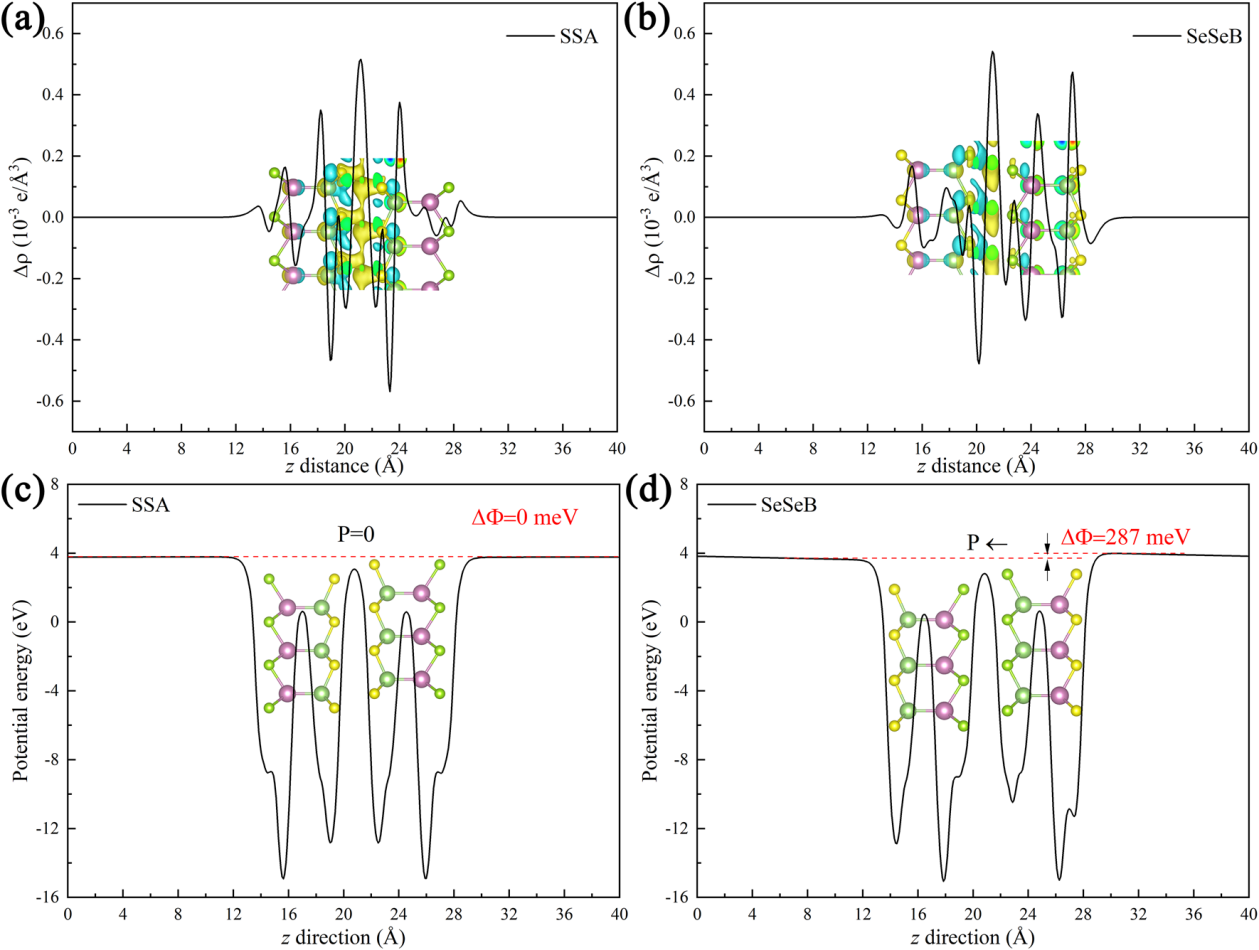
The charge transfer density of SSA (**a**) and SeSeB (**b**) configuration. The average potential energy of SSA (**c**) and SeSeB (**d**) configuration.

To acquire the physical origin of charge transfer and charge redistribution, we calculated the plane differential charge density ($\Delta \rho$) along the *z* direction (see Fig. 5a,b and Fig. S5). In general, the $\Delta \rho$ can be calculated by $\Delta \rho \left( z \right) = \int {\rho_{{SGaInSe{/}SeGaInS}} dxdy} - \int {\rho_{SGaInSe} dxdy} - \int {\rho_{SeGaInS} dxdy}$, where $\rho_{{SGaInSe{/}SeGaInS}}$, $\rho_{SGaInSe}$, and $\rho_{SeGaInS}$ are the charge density in the heterostructure, S-Ga-In-Se and Se-Ga-In-S monolayers, respectively. It is worth noting that the electric dipole in the heterostructure contain two parts: the intrinsic electric dipole in the prime Janus monolayers and the interface dipole caused by charge redistribution^55^. It is found that electron rearrangement mainly occurs in the interspace between two layers, which can induce the built-in electric dipole at the interface. Also, the Bader charge analysis showed that there is a small amount of electron transfer at the interface, resulting in an intrinsic p-n junction. The corresponding transferred charge (from first layer to second layer) for SSA, SSB, SSC, SeSeA, SeSeB, SeSeC, SSeB, SSeA, SSeC and SSeB’ configuration are − 0.0011*e*, − 0.0034*e*, 0.0005*e*, 0.0002*e*, − 0.0066*e*, − 0.0019*e*, 0.0039*e*, 0.0107*e*, 0.0083*e* and 0.0143*e*, which is consistent with the direction of electron transfer in the band arrangement.

In addition, we calculate the electrostatic potential difference $\Delta \Phi$ of the heterostructure, as shown in Fig. 5c,d and Fig. S6. The $\Delta \Phi$ of SSA is 0 meV, which indicates that their left and right work functions are the same because the materials on both sides have the same structure. The $\Delta \Phi$ of SeSeB configuration is 287 meV, which indicates that their left and right work functions are different because the materials on both sides have different structures. Similarly, Guo *at al*. studied the structural and optoelectronic properties of Janus aluminum monochalcogenide (C_2h_-Al_2_XY) (X/Y=S, Se and Te) compounds, and found that the wider atomic size difference leads to a larger ΔΦ^56^. Actually, the dipole in those heterostructures is associated with the stacking order, local configurations, and charge redistribution, and so on.

**Figure 6:**
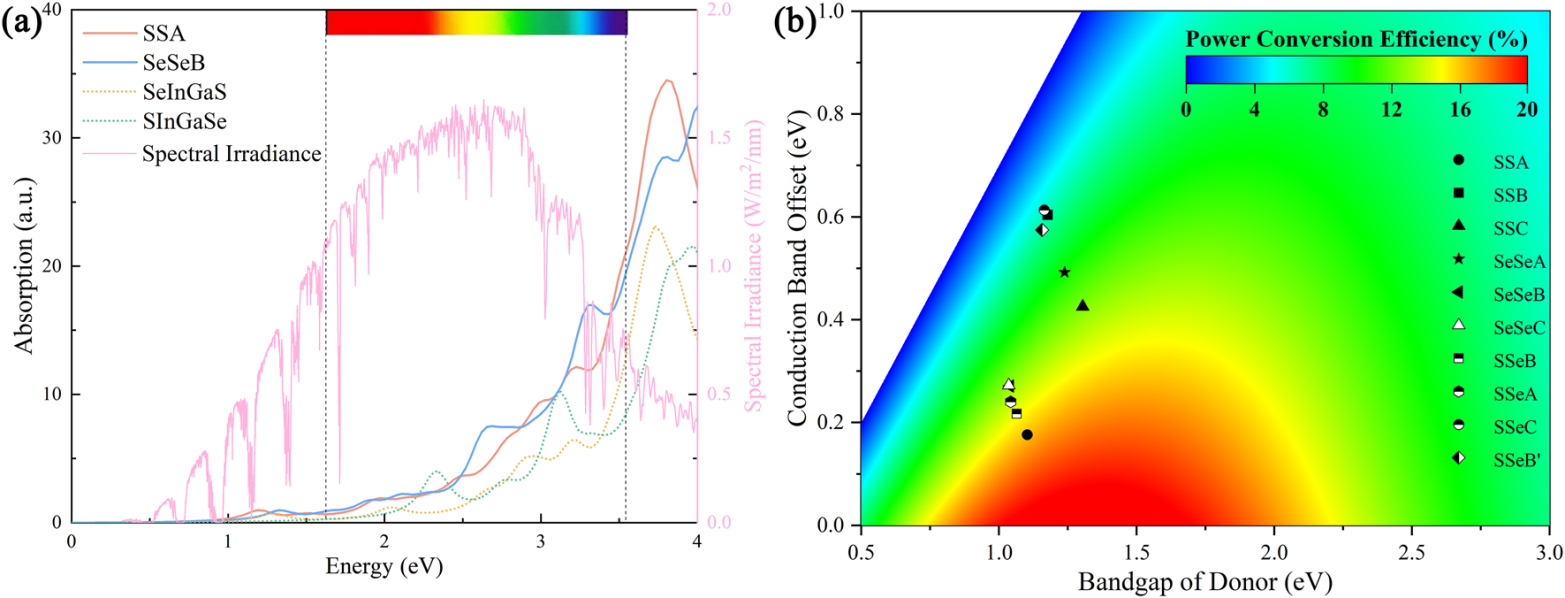
(**a**) The calculated optical absorption coefficients of SSA and SeSeB alignment, which comparison with Janus Se-In-Ga-S, S-In-Ga-Se monolayers and solar spectral radiation. (**b**) The contour plot of power conversion efficiency as a function of donor domain band gap and the CBO for all type II heterostructures.

The optical properties of materials can be revealed by the light absorption governed by the characteristics of electronic band structures, which is the important metric for assessing viability of heterojunctions in the photoelectric devices. Generally, the light absorption coefficient can be deduced from the frequency-dependent complex dielectric function, i.e. $$\alpha \left( \omega \right) = \sqrt 2 {\raise0.7ex\hbox{$\omega $} \!\mathord{\left/ {\vphantom {\omega c}}\right.\kern-0pt} \!\lower0.7ex\hbox{$c$}}\left[ {\sqrt {\varepsilon_{1} \left( \omega \right)^{2} + \varepsilon_{2} \left( \omega \right)^{2} } - \varepsilon_{1} \left( \omega \right)} \right]^{1/2}$$, where $$\omega$$ is the angular frequency, *c* is the speed of light, $$\varepsilon_{1}$$ and $$\varepsilon_{2}$$ denotes the real and imaginary part of the dielectric function. Fig. 6a and Fig. S7 depicts the optical absorption coefficients as a function of photon energy extended from ultraviolet to visible light for in-plane light polarization. As shown, the absorption of heterostructure possess considerable visible light and near-ultraviolet absorption, which are substantially greater than those of Janus Se-In-Ga-S and S-In-Ga-Se monolayer. Meanwhile, the absorption edge of heterostructure shows an obviously red shift owing to the narrow band gap compare with the intrinsic Janus monolayers. Indeed, a narrow band gap of heterostructure with type II band alignment indicates a stronger absorption spectrum because it makes electron and hole pairs easier to generate.

Additionally, the range of incident solar light spectra suggests that these materials have the capacity to absorb sunlight. We carried out more research on the PCE of heterostructures in order to better understand their utilization efficiency for solar light. For heterostructures with type II band alignment, the PCE can estimated by Scharber’s method^57^, i.e. $\eta = \frac{{0.65\left( {E_{g}^{d} - \Delta E_{c} - 0.3} \right)\int_{0}^{\infty } {{{P\left( {h\nu } \right)} \mathord{\left/ {\vphantom {{P\left( {h\nu } \right)} {h\nu }}} \right. \kern-0pt} {h\nu }}d\left( {hv} \right)} }}{{\int_{0}^{\infty } {P\left( {h\nu } \right)d\left( {hv} \right)} }}$, where 0.65 is the fill factor, $E_{g}^{d}$ is the donor band gap, $\Delta E_{c}$ represents the CBO, and $P\left( {h\nu } \right)$ is the AM1.5 solar energy flux. Fig. 6b and Table 3 shows the PCE for all type II heterostructures. The PCE of SSeB and SSA configuration are 12.8%, and 16.2%, respectively. Actually, the PCE of heterostructure is dependent on the band gap and CBO, i.e. a suitable band gap of the donor monolayers for maximum light absorption and a lower CBO for reduced energy loss. Notably, the physical and photoelectric properties of Janus MX monolayers and based heterostructure can be effectively tuned by external factors such as electric field and strain^58,59^. In a word, Janus MX and based heterostructures possess impressive photoelectric conversion capabilities with ultrathin thickness, indicating potential applications in photoelectric systems.

## Conclusion

In summary, the fundamental characteristics of the Janus MX monolayer and ten vdW heterostructures have been systematically studied using first-principles computations. Initially, our findings suggest that the investigated monolayers are semiconducting with band gaps ranging from 1.2 eV to 1.7 eV. Bader charge analysis and electrostatic potential distribution revealed inherent electric field in Janus MX monolayers and based heterostructures. The heterostructures possess higher light absorption coefficient, intrinsic electric field and type II band alignment, which demonstrate that constructing a heterostructure is essential for high photovoltaic performance. Our findings assess the stability and excellent properties of Janus MX monolayers and based heterostructures, recommend them as promising materials for 2D photoelectric applications.

### Supplementary Information

Supplementary Information.

## Data Availability

The data that support the findings of this study are available from the corresponding author upon reasonable request.
