# Supplementary material for: Tunable electronic and photoelectric properties of Janus group-III chalcogenide monolayers and based heterostructures
Source: Sci Rep. 2024 May 10;14:10698. doi: 10.1038/s41598-024-61373-z (PMC11087539; doi:10.1038/s41598-024-61373-z)
Supplement: Supplementary file 1 — Supplementary Information. [file 41598_2024_61373_MOESM1_ESM.doc]

**Supporting Information**

**Tunable electronic and photoelectric properties of Janus group-III chalcogenide monolayers and based heterostructures**

Yipeng Zhao^1^, Qiaolai Tan^2,**^, Honglai Li^3^, Zhiqiang Li^1^, Yicheng Wang^1^, and Liang Ma^1,*^

^1^College of Physics and Electronic Engineering, Hengyang Normal University, Hengyang 421008, China

^2^School of Physics and Electronic Electrical Engineering, Xiangnan University, Chenzhou 423000, China

^3^College of Physics Science and Technology, Hebei University, Baoding 071002, People's Republic of China

*Corresponding author. Email: ml_hw@hotmail.com

**Corresponding author. Email: 492139003@qq.com

**
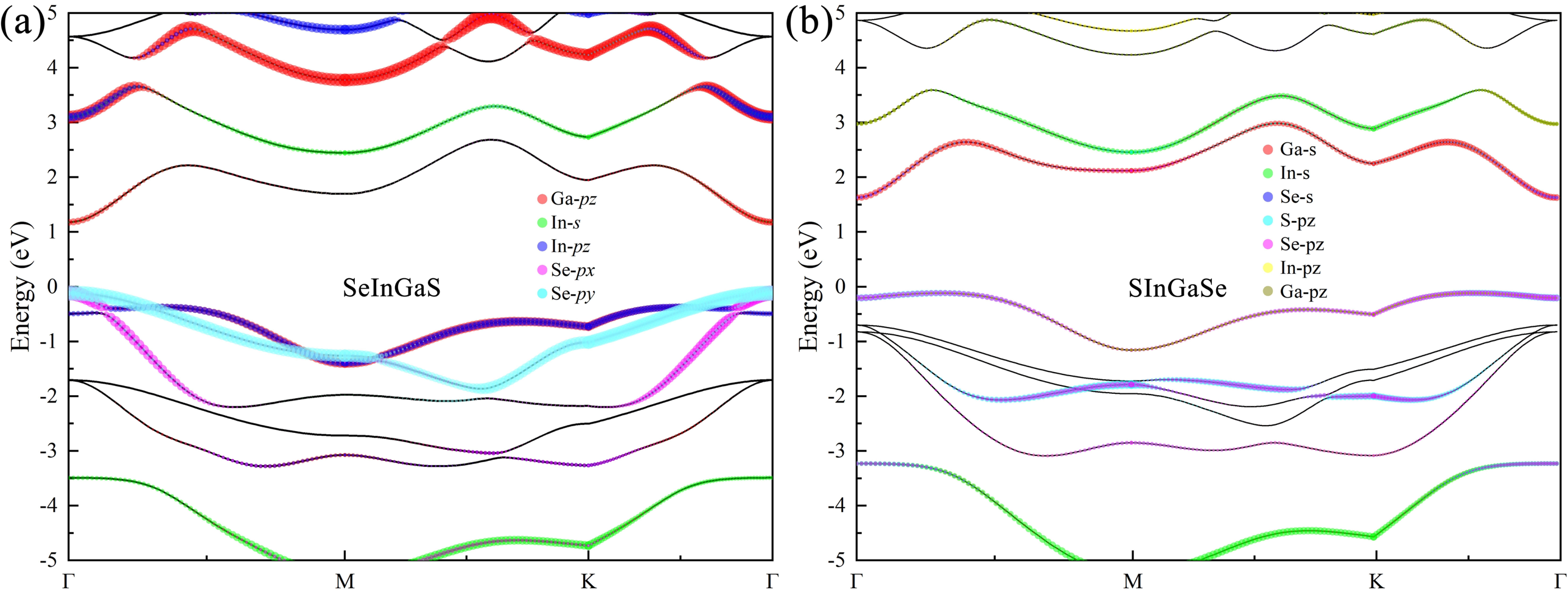
**

**Figure S1.** The orbital-resolved band structures of Janus Se-In-Ga-S (a) and S-In-Ga-Se (b) monolayers.

**

**

**Figure S2.** The band structure and DOS of the 2D Janus group-III chalcogenide for all type II heterostructures.


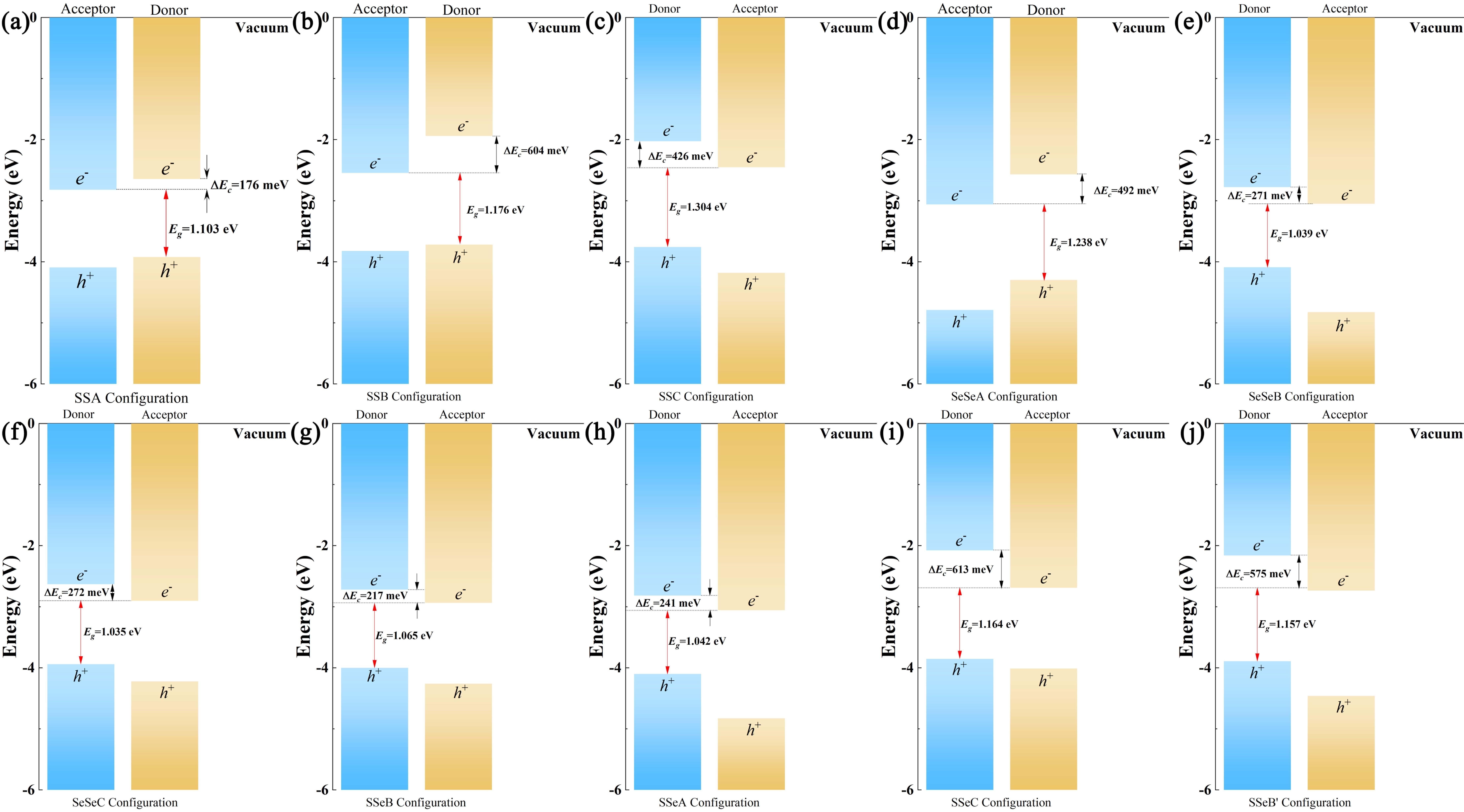


**Figure S3.** Band arrangement of all type II heterostructures.


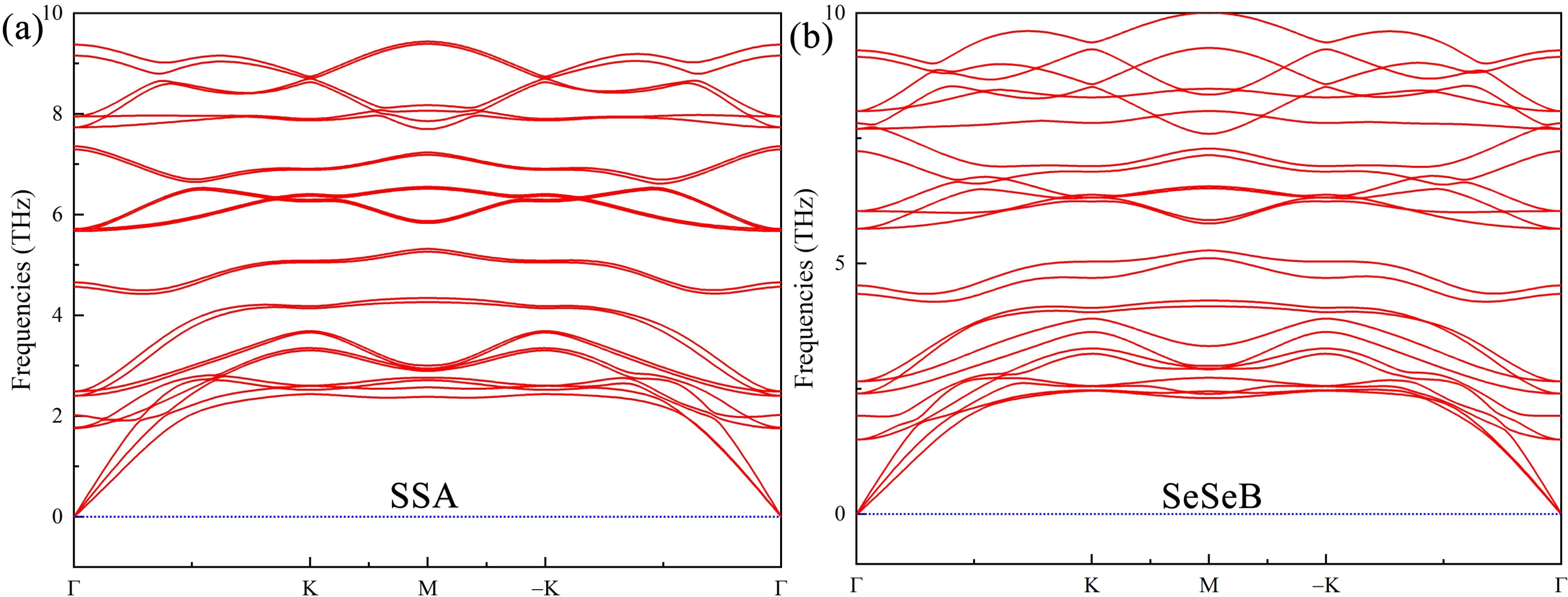


**Figure S4.** The phonon spectrum of SSA (a) and SeSeB (b) configuration.





**Figure S5.** The charge transfer density of all type II heterostructures.





**Figure S6.** The average potential energy of all type II heterostructures.


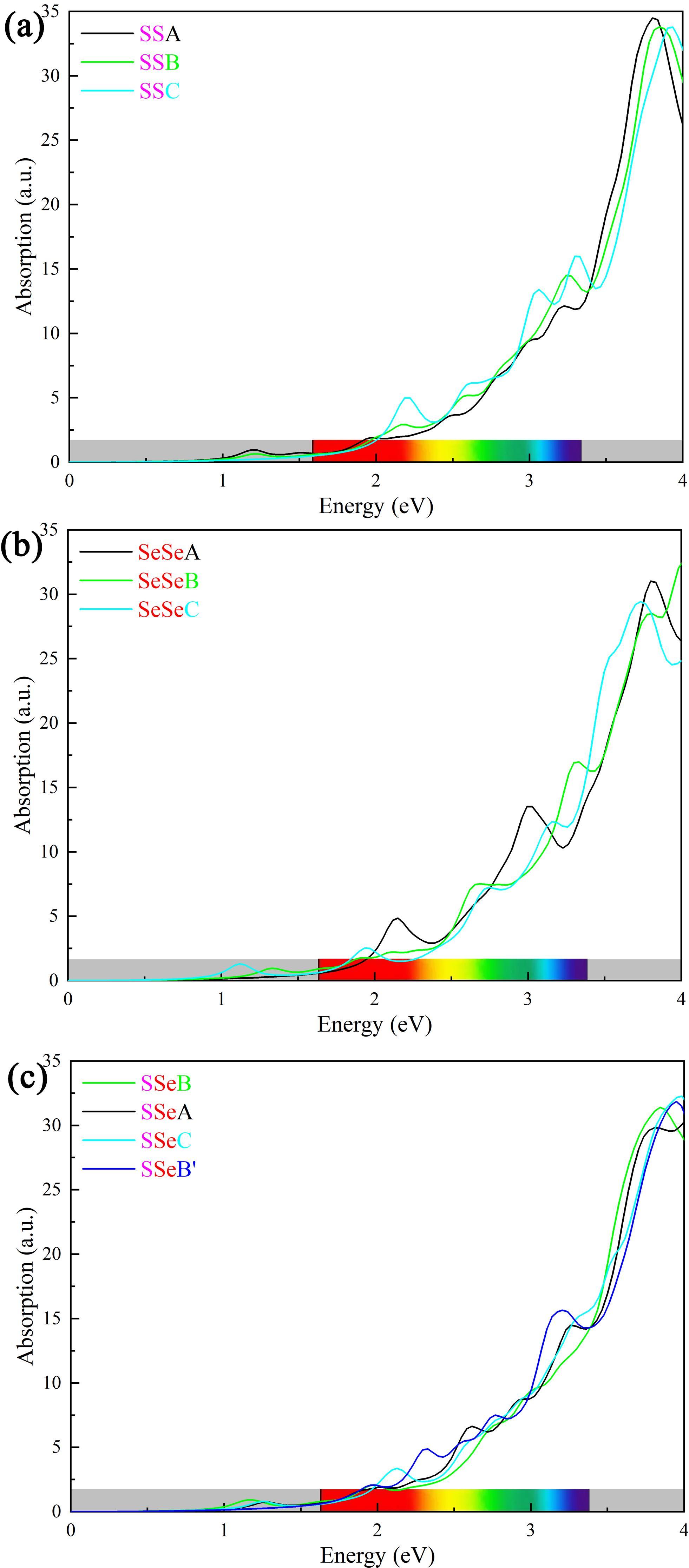


**Figure S7.** The calculated optical absorption coefficients of all type II heterostructures.

**Table S1**. The binding energies of all type II heterostructures for different Stacking type.

| Heterojunction  type | Stacking  type | Energy  (eV) | E-Emin | Heterojunction  type | Stacking  type | Energy  (eV) | E-Emin |
| --- | --- | --- | --- | --- | --- | --- | --- |
| SSA | AA | -31.6886 | 0.0710 | SeSeC | AA | -31.6115 | 0.0673 |
|  | AB | -31.7596 | 0.0000 |  | AB | -31.6787 | 0.0000 |
|  | AC | -31.7571 | 0.0025 |  | AC | -31.67873 | 0.0004 |
|  | AD | -31.6891 | 0.0705 |  | AD | -31.6119 | 0.0668 |
|  | AE | -31.7572 | 0.0024 |  | AE | -31.6752 | 0.0035 |
| SSB | AA | -31.6886 | 0.0710 | SSeB | AA | -31.58982799 | 0.0674 |
|  | AB | -31.7596 | 0.0000 |  | AB | -31.65725104 | 0.0000 |
|  | AC | -31.7571 | 0.0025 |  | AC | -31.6538615 | 0.0034 |
|  | AD | -31.6891 | 0.0705 |  | AD | -31.59027199 | 0.0670 |
|  | AE | -31.7572 | 0.0024 |  | AE | -31.65466074 | 0.0026 |
| SSC | AA | -31.8106 | 0.0692 | SSeA | AA | -31.69172124 | 0.0716 |
|  | AB | -31.8797 | 0.0000 |  | AB | -31.76218152 | 0.0012 |
|  | AC | -31.8792 | 0.0006 |  | AC | -31.76000456 | 0.0034 |
|  | AD | -31.8110 | 0.0688 |  | AD | -31.6920451 | 0.0713 |
|  | AE | -31.8780 | 0.0017 |  | AE | -31.76336031 | 0.0000 |
| SeSeA | AA | -31.8167 | 0.0716 | SSeC | AA | -31.71091686 | 0.0675 |
|  | AB | -31.8879 | 0.0003 |  | AB | -31.77839797 | 0.0000 |
|  | AC | -31.8857 | 0.0026 |  | AC | -31.77687874 | 0.0015 |
|  | AD | -31.8168 | 0.0714 |  | AD | -31.71140269 | 0.0670 |
|  | AE | -31.8882 | 0.0000 |  | AE | -31.77604306 | 0.0024 |
| SeSeB | AA | -31.7139 | 0.0701 | SSeB’ | AA | -31.81550951 | 0.0703 |
|  | AB | -31.7840 | 0.0000 |  | AB | -31.88494584 | 0.0009 |
|  | AC | -31.7815 | 0.0025 |  | AC | -31.88442752 | 0.0014 |
|  | AD | -31.7143 | 0.0696 |  | AD | -31.81594328 | 0.0699 |
|  | AE | -31.7807 | 0.0033 |  | AE | -31.88580941 | 0.0000 |
